# Difficulties in social cognitive functioning among pediatric patients with muscular dystrophies

**DOI:** 10.3389/fpsyg.2023.1296532

**Published:** 2024-01-04

**Authors:** Irune García, Oscar Martínez, Juan Francisco López-Paz, Maitane García, Alicia Aurora Rodríguez, Imanol Amayra

**Affiliations:** Neuro-e-Motion Research Team, Department of Psychology, Faculty of Health Sciences, University of Deusto, Bilbao, Spain

**Keywords:** pediatric MD, social cognition, emotion recognition, theory of mind, general intelligence, behavioral and emotional symptoms

## Abstract

**Introduction:**

Pediatric muscular dystrophies (MDs) are a heterogeneous group of rare neuromuscular diseases characterized by progressive muscle degeneration. A neuropsychosocial approach is crucial for these patients due to associated cognitive, behavioral, and psychiatric comorbidities; however, the social cognitive domain has not been adequately addressed.

**Methods:**

This study aimed to analyze on social cognition performance in a pediatric MD patient cohort. This cross-sectional study included 32 pediatric patients with MD and 32 matched-healthy controls. The Social Perception Domain of the NEPSY-II, the Reading the Mind in the Eyes Test–Child and Happé’s Strange Stories Test were administered. General intelligence and behavioral and emotional symptoms were controlled for to eliminate covariables’ possible influence. The assessments were performed remotely.

**Results:**

Children with MDs performed significantly worse on most of the social cognition tasks. The differences found between the groups could be explained by the level of general intelligence for some aspects more related to theory of mind (ToM) (TM NEPSY-II: *F* = 1.703, *p* = .197; Verbal task: *F* = 2.411, *p* = .125; RMET-C: *F* = 2.899, *p* = .094), but not for emotion recognition. Furthermore, these differences were also independent of behavioral and emotional symptoms.

**Discussion:**

In conclusion, social cognition is apparently impaired in pediatric patients with MD, both for emotion recognition and ToM. Screening assessment in social cognition should be considered to promote early interventions aimed at improving these patient’s quality of life.

## Introduction

Birth- or infancy-onset muscular dystrophies (MDs) are part of the rare pediatric neuromuscular diseases characterized by lesions in the peripheral nervous system (Mah et al., 2016; Dowling et al., 2018; Chikkannaiah and Reyes, 2021). MDs are a heterogeneous group of inherited pathologies whose main sign is a pathological presence of dystrophic muscle, resulting in progressive degeneration and weakness in skeletal muscle (Mercuri et al., 2019; Datta and Ghosh, 2020). The classification of MDs includes: dystrophinopathies (Duchenne and Becker muscular dystrophies–DMD/BMD), myotonic dystrophy, limb–girdle muscular dystrophy (LGMD), Emery–Dreifuss muscular dystrophy and congenital muscular dystrophy (CMD) (Dowling et al., 2018; Mercuri et al., 2019). DMD is the most prevalent form in childhood (Mercuri et al., 2019; Chikkannaiah and Reyes, 2021).

Despite their heterogeneity, pediatric MDs share common clinical features that affect developmental milestones and life expectancy, which lead to lifelong disability in basic activities of daily living (BADL) (Dowling et al., 2018). This disability results in a loss of ambulation and other motor skills disturbances at an early age (Lurio et al., 2015; Birnkrant et al., 2018a). Therefore, early detection of motor developmental delays is critical (van Dommelen et al., 2020; Pasrija and Tadi, 2022). Moreover, associated cardiac and respiratory complications are common in pediatric patients with MDs, representing the main causes of premature mortality (Dowling et al., 2018; Duan et al., 2021; Pasrija and Tadi, 2022).

In addition to motor impairment, this population also experiences difficulties in reaching nonmotor neurodevelopmental milestones (van Dommelen et al., 2020; D’Alessandro et al., 2021; Lee et al., 2022). Regardless of children’s motor development, neuroimaging studies have identified gray and white matter anomalies in cortical and subcortical areas. However, these anomalies are widespread and dependent on the genes involved in each MD subtype (Angelini and Pinzan, 2019; Specht and Straub, 2021). Current research is increasingly focused on the manifestations of the central nervous system (CNS) (Paganoni et al., 2017; D’Alessandro et al., 2021; Specht and Straub, 2021), particularly on associated cognitive, behavioral, and psychiatric symptoms (Darke et al., 2006; Caspers Conway et al., 2015; Lee et al., 2022). Due to the higher prevalence of DMD, most of the literature has focused on characterizing its neuropsychological profile (Perumal et al., 2015; Battini et al., 2018). Intellectual disability is a common feature in pediatric MD diagnoses (Tesei et al., 2020; Mohamadian et al., 2022), resulting in behavioral and psychosocial problems (Panda and Sharawat, 2021; Specht and Straub, 2021). A higher risk of co-occurrence of neurodevelopmental and psychiatric conditions in children and youth with MDs has also been reported compared with the general population (Mohamadian et al., 2022). Additionally, patients with MDs have higher rates of internalizing symptoms (depression and anxiety), autism spectrum disorders (ASDs) and attention-deficit hyperactivity disorder (ADHD) (Colvin et al., 2022; Mohamadian et al., 2022; Pascual-Morena et al., 2022).

While not all pediatric patients with MD present a phenotype associated with these neuropsychiatric syndromes, prototypical behavioral traits are common (Hinton et al., 2006; Fujino et al., 2018; Gosar et al., 2021). It is necessary to consider these prototypical behavioral traits due to their significant involvement in social–emotional development (Darke et al., 2006; Panda and Sharawat, 2021). In fact, children with MDs often show more restricted social and communication skills (Darke et al., 2006; Tesei et al., 2020; Gosar et al., 2021). They also seem to have difficulties in “reading others”; i.e., considering others’ point of view, making inferences, and understanding nonliteral language and nonverbal communication cues, such as facial expressions (Banihani et al., 2015; Poysky, 2018).

These cognitive–emotional difficulties are implicit in the neuropsychological domain of social cognition (SC), which is defined as the cognitive ability to interpret social situations appropriately and act accordingly (Henry et al., 2016; Beaudoin and Beauchamp, 2020). Facial recognition of emotions and theory of mind (ToM) are the two most analyzed subcomponents (de Mello et al., 2023). ToM is described as the metacognitive and socioemotional ability to attribute and understand other’s and one’s own beliefs, emotions, desires, and intentions (Premack and Woodruff, 1978). It is understood as a complex construct divided into two components: affective and cognitive (Shamay-Tsoory and Aharon-Peretz, 2007). Affective ToM is responsible for the emphatic evaluation of the other person’s emotional state whereas cognitive ToM is represented by implications about beliefs, knowledge, and intentions (e.g., Shamay-Tsoory et al., 2007; O’Brien et al., 2011). Moreover, SC is related to other cognitive abilities (e.g., general intelligence) and a necessary skill for good social–emotional functioning. For these reasons, clinicians are increasingly interested in studying SC (McDonald et al., 2023).

A neuropsychological approach is crucial to support children with MDs emotional, cognitive, and social development of Paganoni et al. (2017) and Colvin et al. (2022). Motor disturbances can significantly impair the social interactions of children with disabilities, reducing engagement with peers and negatively affecting their emotional well-being and self-esteem (Kim et al., 2016). The literature also indicates that acquiring age-appropriate motor abilities is essential for the proper development of SC (Leonard and Hill, 2014; Salaj and Masnjak, 2022). Psychological symptomatology has been found to interfere with SC, in which expressly internalizing symptoms are related to a poorer ability to understand others’ emotions (Göbel et al., 2016). Motor disturbances and psychological symptoms are potential risk factors for SC development in children with MDs. Thus, it is necessary to consider SC as an early screening variable, particularly because SC is clearly affected in other similar neuromuscular diseases (Labayru et al., 2018; Serra et al., 2020).

Only two studies have analyzed SC in children with DMD, indicating a worse performance in facial emotion recognition and ToM (Hinton et al., 2007; García et al., 2023). Despite earlier preliminary results (García et al., 2023), there is still insufficient evidence about SC in DMD and other forms of pediatric MDs, suggesting that this is a field of scientific and clinical knowledge to be explored. Given the lack of existing literature in this neuropsychological domain, this study seeks to comprehensively assess SC in a sample of pediatric patients with MDs. For this purpose, the SC performance scores of pediatric patients with MDs and healthy controls were compared after controlling for the general intelligence level and behavioral and emotional symptoms. Likewise, the influence of diagnosis-related variables and physical functionality on neuropsychological performance was also examined.

## Methods

### Participants

A total of 32 Spanish pediatric patients with MDs were included in the study. All participants were aged between 7 and 16 years and were recruited through the following Spanish associations: the Duchenne Parent Project (DPP), the Spanish Federation of Neuromuscular Diseases (ASEM), and the Association of Limb–Girdle Muscular Dystrophy due to Sarcoglycan deficiency (Proyecto Alpha). Table 1 presents sociodemographic and clinical data. A sample of 32 healthy controls, selected by sex and age, was also included. There were no differences between the MD and control groups for sex, *χ^2^*(1) = 0, *p* = 1, or age (*U* = 512.000, *Z* = 0.000, *p* = 1.000).

**Table 1:** Sociodemographic and clinical data of the sample.

	MD group (*n* = 32)	Control group (*n* = 32)
	*N* (%)	*N* (%)
Boys/Girls	27 (84.4%)/5 (15.6%)	27 (84.4%)/5 (15.6%)
Type of pediatric muscular dystrophy		
DMD	21 (65.6%)	-
BMD	3 (9.4%)	-
LGMD	7 (21.9%)	-
LAMA2-CMD	1 (3.1%)	-
Comorbidity with neurodevelopmental disorders		
No one	27 (84.4%)	32 (100%)
Asperger syndrome	2 (6.3%)	0 (0%)
ADHD	1 (3.1%)	0 (0%)
ADHD + High intellectual abilities	1 (3.1%)	0 (0%)
ADHD + Dyslexia	1 (3.1%)	0 (0%)
Treatment		
Glucocorticoid therapy	21 (65.6%)	-
Management		
Musculoskeletal	17 (53.1%)	-
Musculoskeletal + cardiac	4 (12.5%)	-
Musculoskeletal + respiratory	3 (9.4%)	-
Musculoskeletal + cardiac + respiratory	2 (6.3%)	-
Musculoskeletal + cardiac + gastrointestinal and nutritional	2 (6.3%)	-
Musculoskeletal + cardiac + respiratory + gastrointestinal and nutritional	1 (3.1%)	-
Wheelchair need		
Never	19 (59.4%)	-
Intermittent	8 (25.0%)	-
Always	5 (15.6%)	-
	Mean ± SD	Mean ± SD
Age (yrs)	10.31 ± 2.62	10.31 ± 2.62
Onset age (yrs)	3.53 ± 2.46	-
BADL – Barthel’s index	71.72 ± 26.99	98.13 ± 5.04

SD, standard deviation; *N*, number of participants; %, percentage of participants; DMD, Duchenne muscular dystrophy; BMD, Becker muscular dystrophy; LGMD, Limb-girdle muscular dystrophy; LAMA2-CMD, Merosin-deficient congenital muscular dystrophy; ADHD, Attention-deficit hyperactivity disorder; BADL, basic activities of daily living.

Inclusion criteria were: (a) having been diagnosed with MD by a neurologist, (b) being between 7 and 16 years old, (c) having signed an informed consent form through their legal guardians prior to participation in the study, and (d) having Spanish as one of their main languages. The exclusion criteria were: (a) presence of any other diagnosis or sensory deficit that would prevent the application of the tests and (b) being illiterate. These criteria were applied equally to the participants in the control group, except those related to neuromuscular diagnoses.

In addition, 32 parents in both the clinical and control groups participated in the study.

Before its implementation, the study was approved by the Ethics Committee of University of Deusto (ETK-16/21–22) and was conducted following the ethical principles established by the Declaration of Helsinki.

### Instruments

Parents reported clinical and sociodemographic information that was collected prior to the assessment. After a brief interview in which the child is met, the objective of the study is explained and the child’s verbal consent to participate is collected, a trained neuropsychologist administered the neuropsychological tests included in the assessment protocol. The applied instruments had the appropriate psychometric properties and were adapted to the Spanish population. Moreover, the test selection aims to achieve a comprehensive assessment in SC; more specifically, in emotion recognition and ToM (both affective and cognitive).

Instruments completed by parents:

#### Physical functioning

The Barthel Index (Mahoney and Barthel, 1965–Spanish version: Baztán et al., 1993) examines the degree of physical functioning in different BADLs: feeding, washing, dressing, grooming, bowel movements, urination, toileting, transferring chair/bed, ambulation, and stairs. The Barthel’s index score (ranged from 0–100) is the sum of different BADLs scores. It is generally considered the following interpretation for level of dependency (Shah et al., 1989): 0–20 total dependence, 21–60 severe dependence, 61–90 moderate dependence and 91–99 slight dependence.

#### Behavioral and emotional symptoms

The Spanish version of the Child Behavior Checklist for Ages 6–18 (CBCL/6–18) (Achenbach and Rescorla, 2001) corresponds to the ASEBA (Achenbach System of Empirically Based Assessment) multiaxial assessment system for determining behavioral and emotional problems in children and adolescents. It consists of 113 items, grouped into eight syndrome scales: anxious/depressed, withdrawn/depressed, somatic complaints, social problems, thinking problems, attention problems, rule-breaking behavior and aggressive behavior. Response format used 3-point Likert scale (0 = Absent, 1 = Occurs sometimes, 2 = Occurs often). The subscales can be divided into two higher-order scales: internalizing and externalizing problems. By summing up all the problems, a total problems’ score is also provided. A higher score suggests a greater presence of symptomatology.

#### Neuropsychological assessment–general intelligence

Raven’s Progressive Matrices Test (RPM) (2nd edition, Raven et al., 1996) measures general nonverbal fluid intelligence. The person being assessed was given a set of pieces and asked to identify which one completed the figure presented using abstract reasoning. Two versions were used: 1. The Colored Progressive Matrices version includes three blocks of 12 colored items and is designed for children aged five to 11; and 2. The Standard Progressive Matrices version includes five blocks of 12 black-and-white items and can be administered from the age of 12.

The test score is the sum of correct responses.

#### Neuropsychological assessment–social cognition

The Spanish version of the Social Perception Domain of the NEPSY-II (Korkman et al., 2014) assesses SC. The domain consists of two subtests: Affect Recognition (AR), which evaluates the ability to recognize emotions (Happy, Sad, Neutral, Fear, Angry, and Disgust) in different photographs of children. The subtest score is the sum of correct responses (ranged from 0–35); and 2. Theory of mind (TM), is both a cognitive and affective ToM test which includes Verbal and Contextual tasks. The Verbal task assesses the ability to understand others’ ideas, thoughts, and beliefs, as well as figurative language and to engage in imitation. This task uses purely verbal items or items accompanied by a picture (subtest score ranged from 0–22). The Contextual task evaluates the ability to understand and infer the relationship between emotions and the social context (subtest score ranged from 0–6). The Verbal and Contextual task scores are added to calculate the total TM score. The two subtests of the domain can be applied from the age of three to 16 years and 11 months.

The Reading the Mind in the Eyes Test, or Eyes Test–Child version (RMET-C) (Baron-Cohen et al., 2001–Spanish version: Rueda et al., 2013) is a measure of affective ToM. This test includes 28 photographs of people that show part of the face around the eyes. The task involves matching the picture to one of the four words shown that best reflects the thoughts and feelings of the person in the picture. The test score is the sum of correct responses (ranged from 0–28). A score of 18 points is considered a normative value. It can be applied from the age of six.

Happé’s Strange Stories Test (Happé, 1994–Spanish version: Pousa, 2002) is a cognitive ToM test that measures the ability to understand the nonliteral meanings of communication through reading short vignettes. This study applied the version adapted by White et al. (2009), which contains eight story–types, of which the four most used subtests are assessed (misunderstanding, double bluff, white lie, and persuasion). A question is asked after each vignette, and the subject must provide an answer regarding the story’s social context. The test score is the sum of correct responses (ranged from 0–16). A score of 10 points is considered a normative value. It can be administered from the age of seven.

### Procedure

A cross-sectional study was carried out using a convenience sample, considering the characteristics of the clinical population. Recruitment was carried out between November 2021 and November 2022 contacting by email and telephone with the Spanish patient associations, DPP, ASEM and Proyecto Alpha. Each association disseminated the information through a letter to patients who met the inclusion and exclusion criteria of the study. The patients who were interested in participating were informed in more detail about the study. Each participant completed a single individual assessment session of approximately 50 min via videoconference using the ‘Google Meet’ platform to protect the participants’ health during the SARS-CoV-2 pandemic. Before their participation, the patients’ parents signed the proxy consent and completed the tests addressed to them using ‘Google Forms’. The children provided their informed consent verbally. The children in the control group were recruited from Spanish volunteer families who knew about the project through social networks and were interested in participating. After checking the matching criteria, they were proposed to participate in the study. All the assessments were conducted under similar environmental conditions for both groups.

### Data analysis

Statistical analyses were conducted using the statistical software SPSS (Statistical Package for the Social Sciences) version 28.0. The Shapiro–Wilk test was applied prior to the analyses to determine the normal distribution of the variables, which determined that the data did not follow the normal distribution. Descriptive statistics and frequencies were calculated for sociodemographic and clinical data.

For comparison between patient and control groups, the Chi-square and the Mann–Whitney *U* test were used for categorical quantitative variables, respectively. The *r* coefficient (Rosenthal, 1991) was calculated to measure effect size in quantitative variables, where *r* = 0.1 is considered small, *r* = 0.3 medium, and *r* = 0.5 large. Spearman’s *Rho* statistic was performed to analyze the correlation between general nonverbal fluid intelligence and SC scores.

An analysis of covariance (ANCOVA) was carried out to control for the effect of the general intelligence variable on SC performance. Similarly, a multivariate analysis of covariance (MANCOVA) was used to control for the effect of behavioral and emotional symptomatology on SC scores. Finally, multiple regression analyses were carried out to analyze the influence between diagnosis-related variables and physical functionality and the neuropsychological scores of patients with MDs. For this purpose, scores were transformed into *Z*-scores. The level of significance was set at a value of *p* < 0.05.

## Results

**Figure 1:**
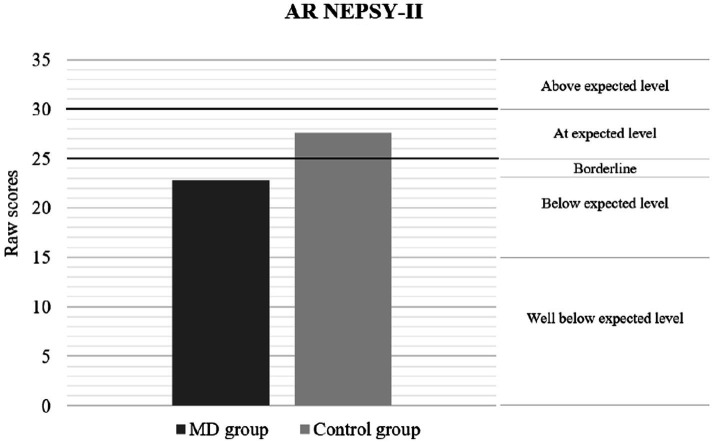
Performance according scaled scores in emotion recognition using AR NEPSY-II. The graph shows that the average performance of the MD group is below what is expected for the average age of the sample. The classification label on the right side of the graph shows how the raw scores correspond to the following scaled scores = Above expected level (13–19); At expected level (8–12); Borderline (6–7); Below expected level (4–5);Well Below expected level (1–3).

**Figure 2:**
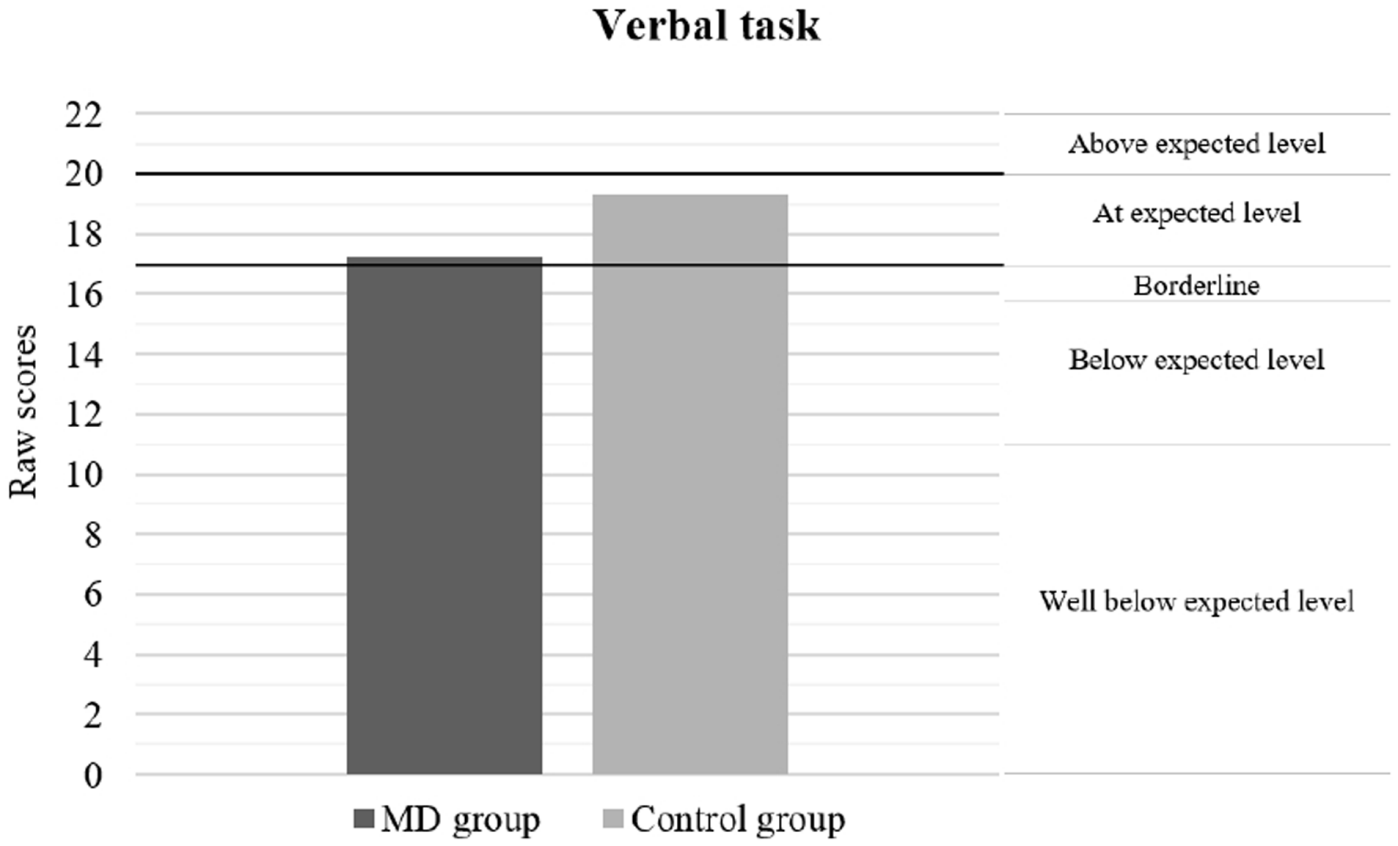
Performance according percentile rank in ToM using verbal task. The graph shows that the average performance of the MD group is at the expected level for the average age of the sample. The classification label on the right side of the graph shows how the raw scores correspond to the following percentile rank = Above expected level (>75); At expected level (26–75); Borderline (11–25); Below expected level (3–10); Well below expected level (≤2).

**Figure 3:**
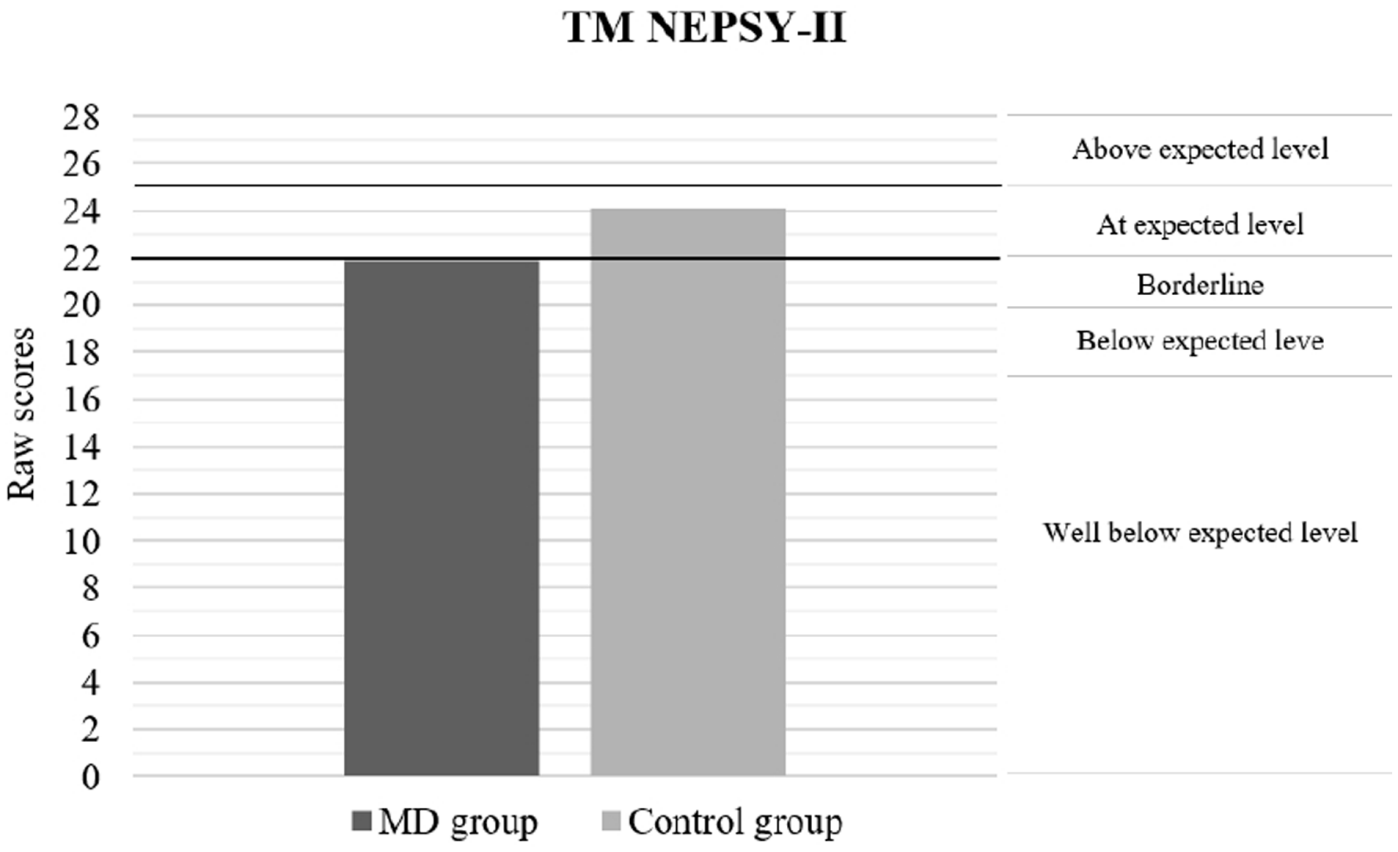
Performance according percentile rank in ToM using TM NEPSY-11. The graph shows that the average performance of the MD group is at borderline level for the average age of the sample. The classification label on the right side of the graph shows how the raw scores correspond to the following percentile rank = Above expected level (>75); At expected level (26–75); Borderline (11–25); Below expected level (3–10); Well Below expected level (≤2).

Table 2 presents the results of the neuropsychological performance between patients with MDs and healthy controls. The findings indicate a significant and lower level of neuropsychological performance by the clinical group, both in general intelligence and SC. Considering the effect size indicator, the magnitude of these differences ranged from medium to large. Figures 1–3 show the differences in the level of performance for the social-cognitive indicators AR NEPSY-II, and Verbal task and total score of TM NEPSY-II. However, no differences were found on the Contextual Task score from the NEPSY-II ToM subtest. Table 2 presents the results of the MD and the control groups in the different behavioral and emotional symptomatology scores measured by the CBCL test. The data show that pediatric patients with MDs have significantly more behavioral and emotional symptomatology. The effect sizes found for these differences ranged from medium to large. However, no significant differences were found between groups on the thought problems and rule-breaking behavior scores of the CBCL.

**Table 2:** Neuropsychological and behavioral and emotional differences between the clinical and control group.

		MD group (*n* = 32)	Control group (*n* = 32)	*U*	*Z*	*p*	*r*
		Mean ± *SD*	Mean ± *SD*				
Social cognition							
Emotion recognition	*AR NEPSY-II*	22.78 ± 5.19	27.59 ± 2.50	215.500	−3.994	<0.001***	0.50
Theory of mind	*TM NEPSY-II*	21.84 ± 3.43	24.13 ± 2.67	313.500	−2.680	0.007**	0.34
	Verbal task	17.22 ± 2.98	19.28 ± 2.16	307.000	−2.782	0.005**	0.35
	Contextual task	4.63 ± 0.98	4.84 ± 1.02	444.500	−.944	0.345	-
	*RMET-C*	16.75 ± 3.72	19.22 ± 2.32	318.500	−2.617	0.009**	0.33
	*Happé’s Strange Stories*	9.56 ± 3.28	12.56 ± 2.03	236.000	−3.733	<0.001***	0.47
Non-verbal intelligence							
RPM		27.69 ± 7.93	33.00 ± 5.95	276.000	−3.174	0.002**	0.39
Behavioral and emotional problems							
*CBCL*	Anxious/Depressed	6.41 ± 4.91	2.56 ± 2.14	253.500	−3.490	<0.001***	0.44
	Withdrawn/Depressed	3.56 ± 3.04	0.88 ± 0.91	187.000	−4.460	<0.001***	0.56
	Somatic complaints	3.87 ± 3.52	0.97 ± 0.93	209.000	−4.175	<0.001***	0.52
	Social problems	5.19 ± 3.51	1.19 ± 1.71	142.000	−.5.048	<0.001***	0.63
	Thought problems	2.37 ± 2.90	0.94 ± 0.91	417.000	−1.639	0.090	-
	Attention problems	6.53 ± 4.71	3.00 ± 3.09	269.500	−3.274	<0.001***	0.41
	Rule-breaking behavior	2.44 ± 2.31	1.41 ± 1.83	374.000	−1.907	0.056	-
	Aggressive behavior	10.06 ± 7.75	3.97 ± 4.53	240.000	−3.664	<0.001***	0.46
	Other problems	4.91 ± 3.15	3.25 ± 2.16	353.500	−2.144	0.032*	0.27
	Internalizing problems	13.84 ± 9.76	4.41 ± 2.77	175.500	−4.529	<0.001***	0.57
	Externalizing problems	12.50 ± 9.79	5.37 ± 6.10	263.000	−3.352	<0.001***	0.42
	Total problems	45.34 ± 28.38	18.16 ± 12.23	184.500	−4.399	<0.001***	0.55

*n*, number of participants; SD, standard deviation; U, Mann–Whitney U test; Z, z scores; r, effect size; AR NEPSY-II, Nepsy-II’s Affect recognition subtest; TM NEPSY-II, Nepsy-II’s Theory of mind subtest; RMET-C, Reading the Mind in the Eyes Test – Child version; RPM, Raven’s Progressive Matrices; CBCL, Child Behavior Checklist. Data are shown in raw scores. ^*^p < 0.05, ^**^p < 0.01, ^***^p < 0.001.

When analyzing the correlations obtained in the total sample, the level of general intelligence measured through the RPM correlated positively with all the scores of SC: AR NEPSY-II (*Rho* = 0.685, *p* < 0.001), TM NEPSY-II (*Rho* = 0.597, *p* < 0.001), TM Verbal task (*Rho* = 0.586, *p* < 0.001), TM Contextual task (*Rho* = 0.309, *p* = 0.013), RMET-C (*Rho* = 0.542, *p* < 0.001), and Happé’s Strange Stories (*Rho* = 0.591, *p* < 0.001).

To control for the influence of general intelligence level on SC performance, an ANCOVA analysis was carried out on those variables for which the previous comparative analysis showed significant differences. Table 3 provides the results obtained by both groups. The differences between groups were eliminated in the TM NEPSY-II (*F* = 1.703, *p* = 0.197), TM Verbal task (*F* = 2.411, *p* = 0.125), and RMET-C (*F* = 2.899, *p* = 0.094) scores, while statistically significant differences were maintained for the rest. The statistically significant differences in SC between groups remained in some scores even after controlling for nonverbal general intelligence.

**Table 3:** ANCOVA and MANCOVA analysis between the clinical and control group.

		MD group (*n* = 32)	Control group (*n* = 32)	ANCOVA			MANCOVA		
		Mean ± *SD*	Mean ± *SD*	*F*	*p*	*η^2^_p_*	*F*	*p*	*η^2^_p_*
Social cognition									
Emotion recognition	*AR NEPSY-II*	−0.48 ± 1.11	0.55 ± 0.54	11.432	<0.001***	0.158	15.883	<0.001***	0.231
Theory of Mind	*TM NEPSY-II*	−0.25 ± 0.97	0.39 ± 0.75	2.234	0.140	0.035	14.142	<0.001***	0.211
	Verbal task	−0.27 ± 0.99	0.42 ± 0.72	3.123	0.082	0.049	13.174	<0.001***	0.199
	Contextual task	-	-	-	-	-	-	-	-
	*RMET-C*	−0.24 ± 0.99	0.42 ± 0.62	3.654	0.061	0.057	6.583	0.013*	0.110
	*Happé’s Strange Stories*	−0.38 ± .96	0.50 ± 0.60	9.545	0.003**	0.135	12.307	<0.001***	0.188

*n*, number of participants; SD, standard deviation; F, ANCOVA/MANCOVA; η^2^_p_, effect size. AR NEPSY-II, Nepsy-II’s Affect recognition subtest; TM NEPSY-II, Nepsy-II’s Theory of mind subtest; RMET-C, Reading the Mind in the Eyes Test – Child version. All data are shown converted into Z scores. *p < 0.05, **p < .01, ***p < 0.001.

Given the differences found in the behavioral and emotional symptomatology reported by parents, a MANCOVA analysis was also performed to control for the effect of this symptomatology on neuropsychological performance in SC. Only indicators that resulted in significant differences (anxious/depressed, withdrawn/depressed, somatic complaints, social problems, attention problems, aggressive behavior, other problems, internalizing problems, externalizing problems, and total problems) were included according to the previous comparative analysis. Table 3 shows how the differences between groups in all scores of SC were maintained once the influence of behavioral and emotional symptomatology was discarded. This means that the statistically significant differences in SC between groups persisted even after controlling for behavioral and emotional symptoms.

Multiple regression analyses assessed the influence of clinical variables on the SC performance of pediatric patients with MDs, specifically those variables related to the diagnosis and physical functionality. The following variables were considered to be predictors of the model: age at diagnosis, wheelchair use and Barthel’s index. This model did not provide significant results for any of the neuropsychological scores of SC analyzed: AR NEPSY-II (*F* = 1.210, *R^2^* = 0.118, *p* = 0.325), TM NEPSY-II (*F* = 1.153, *R^2^* = 0.114, *p* = 0.346), TM Verbal task (*F* = 1.227, *R^2^* = 0.120, *p* = 0.319), TM Contextual task (*F* = 0.518, *R^2^* = 0.054, *p* = 0.673), RMET-C (*F* = 0.374, *R^2^* = 0.040, *p* = 0.772), and Happé’s Strange Stories (*F* = 1.422, *R^2^* = 0.136, *p* = 0.258), or for the general intelligence score RPM (*F* = 0.542, *R^2^* = 0.057, *p* = 0.658).

The results indicated that performance on the different SC scores was not influenced by any clinical variables related to the neuromuscular diagnosis. Likewise, the differences found in the level of general intelligence between the groups were independent of the clinical variables’ characteristics of the MD group.

## Discussion

This study has examined the social cognitive functioning in a group of pediatric patients with MDs and a group of healthy controls, controlling for the effect of the general intelligence level and behavioral and emotional symptoms. The study also analyzed the impact that variables related to diagnosis and physical functionality of children with MDs might have on SC performance.

According to the results, poorer performance was observed in the clinical sample compared with healthy participants on emotion recognition. This is consistent with the studies conducted in boys with DMD showing clear difficulties in emotion recognition (Hinton et al., 2007; García et al., 2023). This difficulty has also been observed in adult patients with MDs, such as myotonic dystrophy type 1 or Steinert’s disease (Labayru et al., 2018; Serra et al., 2020).

Correspondingly, the MD group demonstrated worse ToM performance compared with healthy controls, except in the NEPSY-II’s Contextual task score, where verbal influence is eliminated. In addition, ToM tasks with heavy verbal content seemed particularly difficult for pediatric patients with MDs. The current results are consistent with those of García et al. (2023), who empirically demonstrated that children and adolescents with DMD/BMD exhibited difficulties in ToM tasks. Although there have been no further studies conducted on these pediatric patients to date, ToM was also found impaired in groups of adult patients with neuromuscular diseases (Serra et al., 2016, 2020; Trojsi et al., 2016; Benbrika et al., 2019).

The current findings show SC deficits of pediatric patients with MDs, including both emotion recognition and ToM. Despite the lack of previous scientific studies in this field, supporting literature could explain the obtained results. According to Poysky (2018), children with MDs, particularly with DMD, often show difficulties in “reading others,” including understanding others’ points of view, nonliteral language, and nonverbal communication cues. Moreover, specific neuropsychological deficits (e.g., language and executive functions) identified in some of these diagnoses (Latimer et al., 2017) are strongly related to aspects of SC, especially with ToM (Cumbo et al., 2022). Independently, a crucial aspect to consider in the possible impairment of SC in the pediatric population with MDs relates to difficulties in reaching nonmotor neurodevelopmental milestones (van Dommelen et al., 2020; D’Alessandro et al., 2021; Lee et al., 2022). A growing body of research focuses on the fundamental implications that the early reaching of motor milestones has on other areas of child development (Leonard and Hill, 2014; Salaj and Masnjak, 2022). As the infant grows, motor control development allows the individual to take advantage of learning opportunities by interacting with the physical and social environment. Later on, both fine and gross motor skills are a central component that enables the child to participate in games, particularly to keep up with peers in team games (Leonard, 2016). It is also important to emphasize that early motor disturbances are recognized as a precursor of learning difficulties (Westendorp et al., 2011) and executive control problems (Piek and Pitcher, 2004). Therefore, from this perspective, the child learns about the world by interacting with the environment through motor development and the different senses (Piaget, 1953; von Hofsten, 2004). This interactive process facilitates the cascade development of perceptual, cognitive, and social functioning, while all these skills feed back into the motor domain (Leonard, 2016; Adolph and Franchak, 2017). How motor development impacts on the acquisition of SC has not been studied directly in children with MDs, but there is scientific evidence that confirms these theoretical postulates in other child populations with motor difficulties, such as developmental coordination disorder (Leonard and Hill, 2014; Leonard, 2016; Kilroy et al., 2022). This is linked to more recent theories of “embodied recognition” which hold that a sensory–motor representation of one’s own body is necessary for proper emotional processing and recognition (Niedenthal, 2007; Varela et al., 2017; Lenzoni et al., 2020). This hypothesis may shed some light on the poorer SC performance in children with MDs due to their limitations in movement functionality. This appears to be an opportunity to understand this mechanism in this clinical population better, and further research on this topic is needed (Lenzoni et al., 2020). A recent study has announced that children with intellectual disability are likely to have impairments in imitation ability, which might affect sensory–motor function of simulation processes, leading to poor performance in non–verbal SC task (Ferrari et al., 2023). Finally, these theories provide evidence for other neurodevelopmental conditions with SC involvement and motor dysfunction, which are also more prevalent in the pediatric population with MDs, such as ASD (e.g., Fanghella et al., 2022). While these theoretical postulates do not explain the neurobiological component of SC difficulties in pediatric patients with MDs, they offer new insights into the more restricted social skills and difficulties in the emotional adjustment of these patients (Darke et al., 2006; Colvin et al., 2018; Tesei et al., 2020).

As mentioned before, intellectual impairment may be a common feature among pediatric MDs diagnoses (Tesei et al., 2020; Mohamadian et al., 2022), leading to the emergence of behavioral and psychosocial concerns (Panda and Sharawat, 2021; Specht and Straub, 2021), so it is important to collect this information when conducting neuropsychological assessments (Cotton et al., 2001). Moreover, it has been speculated that there is a strong correlation between nonverbal fluid intelligence and SC, for both emotion recognition (Schlegel et al., 2020) and ToM-related skills (Di Tella et al., 2020; Navarro, 2022). The present study shows a correlation between general intelligence and all SC scores evaluated. Some researchers have previously theorized that social dysfunction in some neuromuscular patients may be due to deficits in general cognitive performance rather than specific SC deficits (Morin et al., 2022). Even so, after eliminating the possible effect of the level of nonverbal fluid intelligence in this study, statistically significant differences between the clinical and control groups were not maintained in the following ToM measures: TM NEPSY-II, TM Verbal task, and RMET-C, indicating that the construction of them items might explain the apparent relationship between these ToM scores and general intelligence. In the case of Verbal task and TM total score of NEPSY-II, it has been hypothesized that general intelligence could be influencing the response to some items, such as those related to metaphors or idioms, in which a greater command of cognitive abilities and specific verbal skills for its successful performance is required. Similarly, some authors have pointed out that RMET-C might not be a “pure” ToM task because of the effect that fluid intelligence has on its realization (Mary et al., 2016; Rosso and Riolfo, 2020). However, the level of intelligence was not a determining covariate for the rest of the SC variables where *a priori* group differences were found, such as facial emotion recognition and ToM tasks, in which compensatory contextual cues are given or involve social knowledge that is much easier to access and more representative of children’s everyday lives (Nawaz et al., 2023). This is a positive finding because, despite the strong correlation between nonverbal fluid intelligence and SC reported in the literature, it appears that cognitive ability might not constrain the development in the specific SC skills of these patients. This raises the possibility that SC development in children with MDs might be different and mediated by other unidentified variables (e.g., social participation opportunities). Thus, researchers and clinicians should continue conducting research in this field to understand and address the difficulties and needs of these patients.

According to behavioral and emotional symptomatology, pediatric patients with MDs presented more internalizing and externalizing problems compared with healthy children of the same age; difficulties related to attention and social problems were also noticeable. These findings are consistent with previous literature (Darke et al., 2006; Panda and Sharawat, 2021; Colvin et al., 2022). Living with a progressive disease such as MD from a young age is not only challenging for the patient’s physical health, but also directly impacts their social development and, consequently, the mental and emotional health of these children (Tesei et al., 2020). Previous research with children with motor impairment has pointed out that psychological symptomatology can interfere with SC abilities. Specifically, internalizing symptoms have been related to a poorer understanding of others’ emotions (Göbel et al., 2016). In the current study, we wanted to test whether the differences in SC between the MD and control groups could be explained by the influence of the more prominent emotional and behavioral symptomatology displayed by patients with MDs compared with healthy subjects. However, these differences remained even after controlling for this symptomatology. This outcome suggests that there is another way to understand the difficulties of these patients in the SC neuropsychological domain, starting with not considering them entirely as a feature of the disease, but rather as a relevant dimension for the comprehensive care of the pediatric patient with MD. Indeed, this is reiterated by the common prototypical social behavioral traits presented by this population (Hinton et al., 2006; Fujino et al., 2018; Gosar et al., 2021).

Similarly, none of the clinical variables related to the diagnosis and physical functionality in the MD pediatric group predicted any worsening of the SC performance measures. This is also consistent with the literature, which emphasizes the nonprogressive nature of these patients’ cognitive difficulties or deficits (Rae and O'Malley, 2016; Tyagi et al., 2020).

Although the psychological symptomatology in pediatric patients with MD and the disease progression do not seem to influence their SC performance, it is essential to continue identifying factors that allow understanding and intervening on these needs. At the same time, it is crucial not to lose sight of the environment and lifestyle that these children adopt as they grow up. Losing functional abilities limits their social participation and fosters isolation (Birnkrant et al., 2018b). Environmental stimulation is indispensable for the development of SC (Soto-Icaza et al., 2015), which leaves children with MDs at a disadvantage and poses another risk factor for their social and cognitive development. Therefore, early neuropsychosocial interventions supported by routine screening protocols in the early stages of development of these patients are essential (Birnkrant et al., 2018a; Tyagi et al., 2020; Gosar et al., 2021) and it is recommended that they be instituted as soon as the neuromuscular diagnosis is made (Darke et al., 2006). It has been shown that psychosocial approaches, including the creation of social support networks and the facilitation of personal pragmatic tools for interaction, reduce the anxiety and depression that these pediatric patients frequently experience (Travlos et al., 2019; Tesei et al., 2020; Pater et al., 2023). In addition, specific neuropsychological training for children in SC has been found to be effective (Hofmann et al., 2016), which favors more functional and social adaptation-related aspects (Hinton et al., 2007; Banerjee et al., 2011) and, ultimately, has an impact on improving quality of life (Maat et al., 2012; Fernández-Sotos et al., 2019).

Despite these findings, this study has several limitations which should be addressed in future research. First, the small number of participants affects the data’s statistical power and extrapolation. Additionally, as DMD is the most prevalent MD, the number of affected patients with the same diagnosis of MD was not proportional (Mercuri et al., 2019; Chikkannaiah and Reyes, 2021). Furthermore, as seen in similar studies with MDs (e.g., Perumal et al., 2015; Piccini et al., 2015; Aden et al., 2023), recruiting participants in research studies is difficult, particularly when they have a rare disease (Pai et al., 2019). Second, the assessment was conducted remotely via videoconferencing due to the restrictions established during the SARS-CoV-2 pandemic (COVID-19). Safety standards were considered while complying with ethical considerations and the validity of the instruments in an online assessment, but the results need to be considered in the conditions in which they were obtained (Loman et al., 2021; Pearson, 2022). Besides, available measures of SC were limited as they depended on the participants’ developmental stage, language, and cultural context. This limits the comparisons with previous studies that included more classically used instruments. Not including an assessment of executive function performance to analyze its relationship with participants’ social cognition functioning is another limitation of this study. Finally, due to the lack of robustness of some non-parametric tests, their parametric versions have been used (ANCOVA, regression analysis), which could to some extent affect the homogeneity of the statistical processing. Therefore, it is prudent to consider these findings as preliminary knowledge due to the lack of studies related to this topic.

This study responds preliminarily to the lack of evidence on the cognitive performance of pediatric patients with MDs (Astrea et al., 2016), with specific regard to the neuropsychological domain of SC. Therefore, it is suggested that future research should conduct new cross-sectional and longitudinal studies to provide further insights into the neuropsychological performance and processing in SC of these patients. It is also important that future research addresses of the present study and can verify the results through replication. This will provide the basis for early intervention and developing specific neuropsychosocial intervention programs not currently available.

In conclusion, this study is the first to extensively analyze the SC performance in patients with different types of pediatric MDs. The preliminary findings demonstrate that SC in children with MDs is impaired compared with healthy-matched children. The level of general intelligence can explain the differences found in SC performance between the groups for aspects related to ToM, but not for emotion recognition. Furthermore, these differences were also independent of behavioral and emotional symptomatology. The SC performance of pediatric patients with MDs was not influenced by any clinical variables related to diagnosis or physical functionality. For this reason, neuropsychological screenings in SC for pediatric patients with MDs are considered necessary to detect specific difficulties and address them early to improve their social development and quality of life.

## Data availability statement

The datasets presented in this article are not readily available because the data that support the findings of this study are available from the corresponding author IG, upon reasonable request. Requests to access the datasets should be directed to irune.garciurquiza@deusto.es.

## Ethics statement

The studies involving humans were approved by Ethics Committee of University of Deusto (ETK-16/21-22). The studies were conducted in accordance with the local legislation and institutional requirements. Written informed consent for participation in this study was provided by the participants’ legal guardians/next of kin.

## Author contributions

IG: Conceptualization, Data curation, Formal analysis, Funding acquisition, Investigation, Methodology, Software, Supervision, Writing – original draft, Writing – review & editing. OM: Conceptualization, Data curation, Investigation, Supervision, Validation, Visualization, Writing – review & editing, Project administration. JL-P: Resources, Supervision, Validation, Visualization, Writing – review & editing. MG: Data curation, Methodology, Software, Supervision, Writing – review & editing. AR: Investigation, Visualization, Writing – review & editing. IA: Investigation, Methodology, Project administration, Resources, Supervision, Writing – review & editing.
